# Maturity-Related Responses to Small-Sided Games in Youth Football

**DOI:** 10.3390/s26010134

**Published:** 2025-12-24

**Authors:** Gonzalo Fernández-Jávega, Ismael Castellano-Galvañ, Manuel Moya-Ramón, Iván Peña-González

**Affiliations:** Sports Research Centre, Department of Sport Sciences, Miguel Hernández University of Elche, Avda. de la Universidad s/n, 03202 Elche, Spain; gonzalo.fernandez02@goumh.umh.es (G.F.-J.); ismael.castellano@goumh.umh.es (I.C.-G.); mmoya@umh.es (M.M.-R.)

**Keywords:** intermittent endurance, biological maturation, 30-15IFT, soccer

## Abstract

**Highlights:**

**What are the main findings?**

- Players improved their intermittent endurance capacity (vIFT) after the 8-week SSG-based training, with no differential adaptation between maturity groups despite clear baseline performance differences.
- External load metrics were comparable across maturity levels, yet less mature players consistently reported higher internal load, indicating maturational differences in the perceptual and physiological response to identical training demands.

**What are the implications of the main findings?**

- SSG-based training can be effectively implemented across heterogeneous maturity groups without compromising aerobic development, supporting its utility as an ecologically valid method for youth football conditioning.
- Coaches should consider biological maturation when interpreting internal load measures, as less mature players may experience disproportionate physiological strain despite similar external loads, with potential implications for load management and injury risk.

**Abstract:**

Biological maturation strongly influences youth players’ physical performance, yet its role in shaping training load responses remains unclear. This study examined how maturation status affects physical adaptations and the relationship between internal load (IL) and external load (EL) during an 8-week small-sided game (SSG) training program in youth football. Fifty-three players were allocated to an experimental group (EG) or control group (CG). EL during SSGs was continuously monitored using 10 Hz GPS units with inertial sensors, while session-RPE quantified IL. Intermittent endurance (vIFT) and 5 m and 30 m sprint performance were assessed before and after the intervention. Players were categorized by years from peak height velocity (PHV). No between-group differences were found in EL variables; however, IL was significantly lower in more mature players. The EG showed a significant improvement in vIFT, whereas sprint performance remained unchanged and the CG showed no improvements. Both maturity groups increased vIFT similarly, with no interaction between maturation status and training adaptation. These findings indicate that SSG-based training effectively enhances intermittent endurance regardless of biological maturity, although less mature players experience higher perceived exertion under equal mechanical demands. Maturation status should therefore be considered when prescribing and interpreting training loads in youth athletes.

## 1. Introduction

The capacity to execute high-intensity runs throughout football matches has been demonstrated as a crucial factor influencing player performance [1]. Pressing actions, exploiting space or supporting moves are some of the high-intensity actions carried out through a game [2]. High-intensity running during matches has increased by a third in the English Premier League across the last decade, thus players must be robust to be able to cope with such demands [3]. The ability to repeatedly perform high-speed runs interspersed with brief recovery periods, termed intermittent running capacity, is therefore believed to be a key performance indicator of soccer. Before reaching high-performance levels, players should have developed a high capacity to cope with these efforts, as it promotes both short-term competitive performance and long-term physical development [4].

Nevertheless, in development stages of young football players, while a well-developed intermittent running capacity is important, the acquisition of technical skills and tactical concepts is also a priority [5,6]. Accordingly, football performance-specialists should consider the most efficient method for conditional, technical and tactical development [7]. The use of SSGs has been established as one of the best integrative approaches for football specific intermittent running capacity [8]. In depth, previous research has shown how different task-constrains (e.g., pitch area, relative area per player, number of players) modulate technical and physical demands within SSG and have scientific evidence to support the attainment of adaptations in running capacity in young players [9]. However, through adolescent development, physiological and morphological changes associated with growth and maturation will be influencing training-adaptation processes [10].

Biological maturation is the developmental process by which humans transition into their adult state, whereas maturity status refers to a unique moment during the path of an individual’s maturation, expressed as age at peak height velocity (PHV) [11]. Maturation leads to physical and physiological changes, including changes in fiber type composition, tendon size and stiffness, and hormonal or anthropometric growth (e.g., increased muscle mass and size) [12]. Differences in biological development can shape how young athletes respond to specific training stimuli. Research examining resistance-training adaptations across adolescence consistently shows that maturational stage plays a meaningful role in determining the dominant mechanisms of improvement [13]. Individuals who are further along in their maturation tend to exhibit more rapid increases in strength, largely driven by growth-related rises in muscle cross-sectional area and the influence of anabolic endocrine activity, which together enhance force-producing capacity [14]. In contrast, athletes who have not yet reached advanced maturation typically rely more heavily on neural adaptations such as improved motor-unit recruitment strategies and refined intermuscular and intramuscular coordination to achieve performance gains [15]. Specifically addressing the influence of biological maturation in endurance-oriented training adaptations, current findings remain inconclusive. While earlier theoretical frameworks proposed the existence of a unique “window of opportunity” for aerobic development around PHV, contemporary research has not consistently supported this assumption [16]. Biological maturation appears to substantially influence how young players perform and interact within game-play situations. More mature players tend to exploit their physical advantages by covering greater distances at higher intensities, engaging more frequently in repeated high-intensity actions, and reaching higher peak speeds during games [17].

A central principle in training theory is the dose–response relationship, which refers to the adaptive effect produced by the interaction between the external load (EL) applied (e.g., the mechanical and locomotor demands imposed on the player) and the internal load (IL) (e.g., the physiological and perceptual responses to that stimulus). EL is now routinely quantified through wearable GPS and inertial measurement systems, which provide continuous, high-resolution tracking of players’ locomotor activity, high-intensity actions, and movement patterns. This technology has become a central tool for monitoring training dose, allowing practitioners to accurately assess the physical demands of specific tasks, tailor session design, and optimize load management [18]. The progression of biological maturation and its related physiological developments can shape how players respond both externally and internally to training demands. Attending to previous statements on this issue, the capacity to tolerate and respond to training stimuli evolves throughout the maturation process [19].

Understanding how biological maturation shapes the responses and adaptations to intermittent endurance training is essential for optimizing the development of youth football players. Although SSG-based programs are widely implemented to simultaneously train technical–tactical behaviors and aerobic fitness, it remains unclear whether players at different maturational stages experience comparable IL, EL, and subsequent performance adaptations. Therefore, the aims of this study were to examine the influence of maturity status on physical adaptations and on the relationship between IL and EL in a training program based on SSGs in youth football, using wearable GPS and inertial sensors.

## 2. Materials and Methods

### 2.1. Study Design

A pre–post intervention design was developed to determine the effects of an SSG-based program on the intermittent endurance of young soccer players. The SSG-based training program was integrated into the players’ regular soccer training regimen for 8 weeks. It was characterized by a short volume and an increase in intensity over the duration of the program. To evaluate the effects of this program, the players’ final velocity at the intermittent fitness test (vIFT) was measured using the 30:15 IFT. Additionally, sprint times for 5 m and 30 m distances were assessed. Players were initially tested one week before starting the program (testing session 1 [TS1]). After the 8-week program, the players were re-evaluated (testing session 2 [TS2]) to determine the impact of the training program.

### 2.2. Participants

Fifty-three young (U14-U16) male soccer players from four different teams of a youth soccer academy participated in this study (age = 14.16 ± 0.91 years; PHV = 0.40 ± 1.00 years; body mass = 57.00 ± 10.83 kg; body height = 167.05 ± 8.10 cm). Two teams were randomly selected for the experimental group (EG) (n = 32), while the other two teams served as the control group (CG) (n = 21). All players in the EG (n = 32) completed at least 80% of the training program. Twenty-eight players completed 100% of the training sessions, two players missed one session (93.75% attendance), one player missed two sessions (87.5% attendance), and one player missed three sessions (81.25% attendance). Players who sustained any injury during the intervention period were excluded from participation and subsequent analysis. Participants and their parents or guardians were informed about the aims and protocols of the study. Both the players and their parents/guardians signed an informed consent form before participating in the study. The study protocol was approved by the ethical guidelines of the hosting institution (Reference number DPS.EC.01.17).

### 2.3. Maturity Assessment and Aggrupation

The most used indicator of somatic maturity status in sports is the “maturity offset,” or the years from/to peak height velocity (PHV) [20]. This is considered a benchmark for the maximal rate of growth in height during adolescence, which typically occurs around fourteen years old in boys and around twelve years old in girls [11]. By predicting the years from/to PHV [21], investigators can obtain accurate data on a young athlete’s maturity status, which is particularly precise for boys aged 12 to 16 years with average maturation [20]. For the analyses, players were categorized into two maturity groups (MG) based on their years from/to PHV: MG1 (players with a PHV ≤ 0.50, n = 26) and MG2 (players with a PHV > 0.50, n = 27).

### 2.4. Testing Procedures

#### 2.4.1. Anthropometrics

Players’ body height and sitting height were measured using a fixed stadiometer (SECA Ltd., Hamburg, Germany, ±0.1 cm). Body weight was measured with a digital body composition monitor (Tanita BC-601 Ltd., Tokyo, Japan, ±0.1 kg).

#### 2.4.2. The 5 m and 30 m Sprint

Players performed two 30 m linear sprints, and the times at 5 m and 30 m were recorded using photoelectric cells (Witty System, Microgate, Bolzano, Italy). The best time for each distance was used for further analysis. Players started the sprint from a standing position, 30 cm before the starting timing gate, and were encouraged to perform each sprint at maximal effort.

#### 2.4.3. The 30-15 Intermittent Fitness Test (IFT)

Players’ intermittent endurance capability was measured using the 30-15 IFT [22]. This test consists of 30 s of running a 40 m distance with changes in direction, interspersed with 15 s of walking for recovery. The initial velocity of the test is 8 km·h^−1^, with 0.5 km·h^−1^ increments in velocity after each 30 s stage. The test concludes when the player is unable to maintain the running speed. The velocity of the last completed stage was recorded as the final velocity in the 30-15 IFT (vIFT).

#### 2.4.4. Training Program

Both groups (EG and CG) performed two football training sessions per week. Since all teams included in this study belonged to the same academy, and despite not performing the same tasks in each training session, the conditional contents of the tasks were planned by the academy’s coordinator and did not differ between teams. This approach assumes that any effects of these trainings on players’ performance would be equal. Additionally, an agreement was reached with the academy’s coordinator and coaches that no analytical work focused on improving endurance (e.g., running-based tasks like HIIT) could be conducted. However, it is acknowledged that the football-specific training tasks themselves contain a cardiovascular component that was not controlled for in this study.

Players in the EG completed a SSG protocol twice weekly, performed at the beginning of each scheduled training session, while the CG performed their usual training without any specific tasks aimed at improving intermittent endurance. This protocol was developed in accordance with [9] and did not suppose an extra volume compared to the CG. On the first training day of the week (match day + 2), a short-format SSG (SSSG) was implemented, with formats ranging 3 vs. 3 to 5 vs. 5, whereas on the second day (match day −3), players engaged in a large-format SSG (LSSG), with formats ranging 7 vs. 7 to 10 vs. 10. SSG progressed in task duration from 3 × 3′/1.5′ (SSSG) and 2 × 5′/1.5′ (LSSG) in weeks 1 and 2 to 3 × 4′/2′ (SSSG) and 2 × 6′/2′ (LSSG) in weeks 3 and 4 with a space per player of 100 m^2^. From week 4 onwards, the space per player was increased to 150 m^2^ with the same progression in task duration in weeks 5–6 and 7–8. The objective of the task was to accumulate more passes than the opposing team. Neither goalkeepers nor goals were included. All training sessions were held at the same venue and time for all participating teams throughout the intervention. Before the SSG task, players performed a standardized warm-up consisting of 5 min of low-intensity activities (e.g., steady-state running and joint mobility exercises), followed by 3 min of dynamic stretching. Finally, players performed 2 min of the SSG (without recording data) as a specific part of the warm-up. During SSG task, balls were replaced immediately when they crossed the sideline or finish line. Coaches gave verbal encouragement to players during the exercises. Regular team training included a warm-up, technical drills (e.g., passing, ball control, shooting) and tactical exercises (e.g., positional play, team organization or game simulation). This structure and training tasks were the same for both EG and CG. No additional strength, conditioning, or individualized training was prescribed to the CG beyond the regular team training.

### 2.5. Tracking Variables

The physical demands of players during SSG tasks were assessed using a wearable device (“OLIs”), which recorded data at a sampling frequency of 10 Hz (Oliver IMU^®^, Barcelona, Spain). At the start of each training task, OLIs were affixed to all players. Unlike other comparable devices, the OLI is positioned on the back of the player’s dominant leg, enabling the measurement of both the frequency and velocity of ball contacts. Validity and reliability of this tracking system have been reported previously [23]. The variables extracted from the OLI device included the following:

- Maximal velocity (km·h^−1^): The highest recorded movement velocity achieved by the player during the match.
- Total distance (m): The total meters covered by the player during the match.
- Walking (m): Distance covered while walking (<6 km·h^−1^).
- LI running (m): Distance covered at low intensity running (6–12 km·h^−1^).
- MI running (m): Distance covered at moderate-intensity running (>12–18 km·h^−1^).
- HI running (m): Distance covered at high intensity running (>18 km·h^−1^).
- Ball contacts (n): Total number of contacts with the ball.
- LI ball contacts (n): Number of low-intensity (ankle velocity < 11 m·s^−1^ but acceleration > 20 G) contacts with the ball.
- MI ball contacts (n): Number of moderate-intensity (ankle velocity 11–15 m·s^−1^) contacts with the ball.
- HI ball contacts (n): Number of high-intensity (ankle velocity > 15 m·s^−1^) contacts with the ball.
- Shot speed (km·h^−1^): Maximum velocity reached during a ball strike in the match.
- Dribbling distance (m): Distance covered in contact with the ball.
- MI accelerations (m): Distance covered with moderate-intensity accelerations (>2 to 3 m·s^−2^).
- HI accelerations (m): Distance covered with high-intensity accelerations (>3 m·s^−2^).
- MI decelerations (m): Distance covered with moderate-intensity decelerations (<−2 to −3 m·s^−2^).
- HI decelerations (m): Distance covered with high-intensity decelerations (<−3 m·s^−2^).

### 2.6. Statistical Analysis

A Shapiro–Wilk test was used to assess the normal distribution of the data for each variable. An initial independent samples *t*-test was conducted to evaluate the initial differences in physical performance between players with different maturity statuses. Another independent samples *t*-test was performed to assess the initial differences between the EG and CG. A paired-sample *t*-test was used to determine the pre–post differences in physical performance within each group. The differences in the tracked variables between the SSSG and LSSG were assessed with a paired-sample *t*-test, while a final independent samples *t*-test was used to evaluate the differences in tracking variables between players with different maturity statuses for each SSG format.

A repeated measures (RM) ANOVA with a Bonferroni post hoc test was conducted to assess the physical performance improvements (pre–post) according to the maturity group. The Effect Size (ES) at a 95% confidence interval (CI) was calculated for each pairwise comparison using Hedges’ g value, following the recommendations of Lakens [24]. The effect sizes were interpreted as trivial (<0.24), small (0.25–0.49), moderate (0.50–0.99), and large (>1.00) [25]. The relationship between players’ improvement in physical performance and their training loads (tracked variables and RPE) was analyzed using Pearson’s correlation coefficient (r). This was interpreted as trivial (<0.09), small (0.10–0.29), moderate (0.30–0.49), high (0.50–0.69), very high (0.70–0.89), and almost perfect (>0.90) [25]. All statistical analyses were carried out using Microsoft Excel (Microsoft, Seattle, WA, USA) and JASP software (JASP Team, Version 0.17.3), with the level of significance set at *p* < 0.05.

## 3. Results

When comparing the physical performance evaluated in the pre-assessment session for the entire sample, players with more advanced maturity status performed better than those with a less advanced maturity status (Table 1). After creating the EG and CG, the independent samples *t*-test showed that both groups had no initial differences in physical performance values (Table 2). The EG improved its physical performance after the 8-week training program (5 m sprint: Pre = 1.04 ± 0.05, Post = 1.05 ± 0.07; t = −0.76; *p* = 1.00; ES [95% CI] = −0.10 [−0.46; 0.26]; 30 m sprint: Pre = 4.48 ± 0.23, Post = 4.50 ± 0.25; t = −0.66; *p* = 0.61; ES [95% CI] = −0.03 [−0.16; 0.10]; vIFT: Pre = 18.92 ± 1.28, Post = 20.08 ± 1.35; t = −5.70; *p* < 0.01; ES [95% CI] = −0.77 [−1.19; −0.35]), while the CG did not improve its performance after the 8 weeks (5 m sprint: Pre = 1.04 ± 0.10, Post = 1.06 ± 0.09; t = −2.37; *p* = 0.13; ES [95% CI] = −0.37 [−0.81; 0.07]; 30 m sprint: Pre = 4.53 ± 0.46, Post = 4.61 ± 0.48; t = −3.87; *p* = 0.01; ES [95% CI] = −0.22 [−0.39; −0.05]; vIFT: Pre = 18.48 ± 1.63, Post = 18.45 ± 1.91; t = 0.10; *p* = 1.00; ES [95% CI] = 0.02 [−0.45; 0.48]).

The training program for the EG consisted of SSSG and LSSG. The differences in tracking variables between these two types of tasks are presented in Table 3. Additionally, the differences in these parameters between players with different maturity statuses for each type of SSG are shown in Table 4. The RM ANOVA showed an improvement in vIFT for both maturity groups (MS1 and MS2) after the training period (F = 21.33; *p* < 0.01), but there was no interaction effect between the time variable and maturity group (F = 0.01; *p* = 0.98). The 5 m and 30 m sprints did not show changes after the 8-week intervention period (F = 0.40–0.42; *p* = 0.53), and there was no interaction effect between the time variable and maturity group (F = 0.15–0.25; *p* = 0.62–0.70). The pairwise differences (pre–post) shown by the post hoc analysis are presented in Table 5 for the EG. The CG showed no pre–post differences (*p* > 0.05) in any of the analyzed physical performance variables across maturity groups. Pearson’s correlation analysis showed that none of the tracking variables nor RPE in any SSG format correlated with the percentage improvement in vIFT, nor with the percentage improvement in the 5 m and 30 m sprints.

## 4. Discussion

The aim of this study was to analyze the effects of an 8-week training program based on SSGs on the physical performance of youth soccer players, considering their biological maturation status. The findings provide valuable insights into how young players with different maturation levels respond to game-based training in terms of performance, EL, and perceived exertion.

### 4.1. Influence of Maturation on Initial Performance

The initial performance evaluation shows a superior physical performance among players with an advanced maturity status. Players with higher maturity status exhibit greater performance in running capacities, both in speed-related qualities acceleration (5 m sprint) and linear velocity (30 m sprint) and intermittent running endurance (vIFT). These results align with the previous evidence of the strong influence of maturity status on physical capacities. Many recent studies have consistently reported higher physical performance among young football players with advanced maturity status, showing superior outcomes in neuromuscular capacities, such as sprint or jump test as well as in intermittent running capacity [26,27,28].

The enhanced force application and speed exhibited by young athletes during the maturation process can be attributed to structural changes in muscles (e.g., fascicle length, muscle hypertrophy, tendon stiffness), neural improvements (e.g., fiber recruitment, increased pre-activation, reduced agonist–antagonist co-contraction), and other changes (e.g., hormonal or metabolic) [16,29]. The maturity-related adaptations influencing intermittent running are less clearly defined. More mature players may possess larger ventricular size and greater stroke volume. This may result in improved cardiac output, higher hemoglobin concentration, and enhanced oxygen transport and utilization [30]. As for that, less mature players may compensate for this handicap with higher heart rates [31]. Doncaster et al. [32] found that pre-PHV soccer players showed superior aerobic running economy compared to circa-PHV players. The study also revealed that whilst absolute measures of peak oxygen uptake were higher in circa-PHV players, values were similar between groups when expressed relative to body mass and fat-free mass. While 30-15 IFT is a reliable and frequently employed test for intermittent running capacity, it relies substantially on peak running speed, particularly in its later stages. As a result of this reliance on neuromuscular pathways, determinant for running speed, 30-15 IFT performance is not completely determined by aerobic capacity. The mentioned maturity-related changes may elicit higher absolute running speed outputs for advanced in maturity status players, which will consequently result in lower relative demands during the test. Running at higher relative speeds increases metabolic cost and energy expenditure, in addition to a higher recruitment of type II muscle fibers, which are less efficient and underdeveloped in less mature players, which present a predominantly type I muscle fiber composition and relies mainly on oxidative pathways for energy production [33,34,35]. As for that, Pre-PHV players may struggle to maintain necessary speeds due to poorer baseline sprint capacity, rather than fatigue or aerobic limitations.

These results underscore the substantial impact of maturational development on running performance, accentuating the physiological, metabolic, and biomechanical advantages of more mature players. Recent literature shows how this physical outperformance is also manifested in the match-running performance of young football players, where players with advanced maturity status covered more distance at high speeds [17]. This association may reinforce what should particularly be focused on during the selection processes to avoid excluding less mature players whose physical and footballing potential may only become apparent after puberty.

### 4.2. Effects of SSG’s Intervention on Physical Performance

The post-intervention analysis revealed significant improvement in vIFT following the 8-week SSG-based training in the EG, whereas sprint performance (5 m and 30 m) remained unchanged; meanwhile, the CG did not show physical performance improvements.

These results support previous research indicating that SSGs are effective in enhancing aerobic capacity due to their intermittent nature, frequent changes in direction, and game-specific intensity [35]. The use of SSG as a training method in youth soccer has gained considerable popularity due to its ability to simultaneously develop technical–tactical skills and physical capacities comparable to specific running alternatives as HIITs [36]. In addition to our findings, the recent literature has postulated SSG as a valuable alternative to running HIIT achieving similar improvements in maximal or peak oxygen uptake and variables related to running performance [37]. Unlike traditional HIIT, which typically involves structured, predictable running at controlled intensities, SSGs embed physical load within the context of decision-making, game constraints and ball involvement. This integrated approach makes SSGs more engaging and sport-specific, potentially enhancing motivation and adherence, particularly among young people without losing the opportunity to enhance the players’ running capacities [38,39].

Nevertheless, a key limitation identified in this study was the absence of improvements in linear sprint performance after the SSG intervention, a result not uncommon in the literature. Certain research reported that SSGs may not simulate high-intensity and sprint demands of competition [40]. However, these acknowledgments should be moderated, as task constraints may be limiting high-intensity action requirements of players hindering the development of this attribute [41]. Therefore, while SSGs are a valuable and time-efficient tool for developing aerobic fitness in youth players, they should be strategically integrated with complementary methods as transition situations with specific constraints to promote high-speed running (e.g., large pitch dimensions, directional play, minimal tactical restrictions) [42].

### 4.3. Tracking Training Load in SSSG and LSSG

The comparison of tracking variables in external and internal metrics between SSSG and LSSG revealed significant differences in the locomotor GPS-tracking variables and perceived effort between each task format. SSSG imposed a substantially higher locomotor load, evidenced by significantly greater total distance covered and higher volumes of moderate and high intensity accelerations and decelerations. Moreover, SSSG promoted a more demanding technical environment, as reflected by the significantly greater number of total ball actions, together with larger dribbling distances.

In contrast to our findings, Owen et al. [43] reported greater physical demands in LSSG compared to SSSG formats. This discrepancy is likely explained by differences in how playing space was manipulated across studies. In their work, the increase in game size was accompanied by a substantial expansion of the relative playing area per player, which naturally enabled and required greater running activity. In our study, however, both SSSG and LSSG were designed with a comparable area per player; as a result in SSSG there are fewer passing options, combined with the fact that there is more pressure applied on the player in possession of the ball, increasing the need of moves and technical actions. Meanwhile, in line with our results, Clemente et al. [44] concluded that when the number of players is reduced in SSG games, particularly high-intensity actions are increased, and this might be the reason for the moderately greater playing loads reported in their study.

The significant higher RPE reported during SSSG indicates that players perceived this format as more physiologically and cognitively demanding. This differences in IL are in line with previous studies, Silva [45] shows in their intervention that the reduction in the number of players in SSGs without changing pitch dimensions increased exercise intensity (%MHR), and Hill-Haas [35] reported that SSSG formats decrease in size and when relative pitch area remains constant, overall physiological and perceptual workload increased. Relating these results with the technical requirement differences between tasks, according to dribbling the ball increases HR responses in football players significantly, which linked to the larger dribbling distances covered in SSSG could explain the intensification in perceived exertion during this task format. These results highlight the relevance of the number of players SSG tasks demand, reducing player numbers, even when relative space remains constant, systematically increases technical requirements, and elevates both EL and IL demands, underscoring the importance of carefully manipulating player ratios when designing training tasks aimed at targeting specific performance outcomes.

Furthermore, a particularly noteworthy result is that none of the GPS-tracking variables (e.g., distance covered, average speed, high-intensity actions), nor the RPE, correlated with improvements in vIFT. This absence of association suggests that short-term variations in EL and IL during SSG-based training may not directly determine the magnitude of aerobic adaptations in youth players. The complex blend of technical, tactical, metabolic, and decision-making demands inherent to SSGs likely produced a potent stimulus across all players, ultimately driving training adaptations.

### 4.4. Maturity-Related Responses to SSG

The comparative analysis of external GPS-derived load metrics revealed no significant differences between MS1 and MS2 players across either SSSG or LSSG formats. In contrast, the internal training response exhibited marked variations depending on the grade of biological development.

Despite achieving EL metrics comparable to their peers with higher maturity status, less mature players perceived higher subjective effort, implying that while the mechanical demands of the SSG training task were equal, the training stimulus was perceived as more demanding for these less mature players. These findings align with Salter [46], who also reported heightened psychophysiological responses in pre-PHV players under identical locomotor requirements. In addition, during this study physical response to fatigue was analyzed. Likewise, their study found larger performance decrements in less mature players as fatigue developed. In turn, similar maturity-related discrepancies in IL have been reported in youth football [19] as well as in interventions employing running-based HIIT, where RPE and IL differed significantly across maturity groups despite uniform EL prescription [47].

Complementing this evidence, Garcia-Ceberino [48] found that although variables such as distance per minute, maximal and average speed, and heart rate did not differ significantly across levels of biological maturity, more mature players engaged in a greater number of high-intensity actions, accelerations, and decelerations during SSGs and training matches. Moreover, they reported a negative correlation between player load per minute and maturational age, suggesting that more mature players manage neuromuscular load more efficiently. As in line with this impairment in external-internal training load between maturity groups, after analyzing 4000 football training sessions, Salter [49] observed that a 5% increase in PAH implied seven sRPE units every session, resulting in a cumulative difference of up to 40% in perceived load over a season. These variations in IL perception with adulthood may be due to both physiological and perceptual factors. Less mature players may have less efficient running economy, lower neuromuscular efficiency, and higher relative physiological strain, making the same EL feel more difficult. From a practical perspective, this imbalance in EL–IL relationships within maturity-heterogeneous squads may place less mature athletes closer to their physiological ceilings, altering the dose–response cycle and potentially increasing injury risk. Bio-banding appears to mitigate this issue, as pre-PHV players report lower perceived exertion without changes in EL when grouped according to biological development [50].

The training intervention demonstrated a marked enhancement in vIFT for both maturity groups (MS1 and MS2) post-intervention, though no interaction effect was observed between the time variable and maturity group. The lack of a significant interaction between time and maturity status suggests that the relative training adaptations were comparable across maturity levels, although physiological differences between maturity groups could theoretically favor superior adaptations in more mature players [51]. However, the previous literature showed that improvements in aerobic performance are driven predominantly from central cardiac adaptations (increased stroke volume (SO) and enhanced ventricular filling) rather than from maturational or hormonal factors [33,50]. Supporting this interpretation, Buchheit [52] provides a valuable physiological framework to interpret why maturity status may not modulate training-induced improvements in intermittent aerobic performance. Their results showed that there were no “maturity × time” interactions for any of the hemodynamic or autonomic measures (e.g., SO, cardiac output), and the temporal patterns of post-exercise hemodynamic and autonomic recovery were not influenced by maturation. Thus, the similar improvements in vIFT observed in both maturity groups in our intervention could be explained by this shared autonomic strain, which ensures a sufficiently potent stimulus to induce central aerobic adaptations regardless of maturity status.

Extrapolating this reasoning, it is plausible that the intermittent stimulus imposed by SSG-based training promotes adaptations that are relatively independent of biological maturity, even in players who have surpassed their PHV. The mechanisms underpinning intermittent running improvements (e.g., central hemodynamic adjustments, improved oxygen delivery, and enhanced repeated-effort tolerance) may be similarly trainable across maturity levels when the training stimulus does not rely heavily on neuromuscular or hormonal factors.

These results are consistent with those obtained during a running-based HIIT intervention, with comparable methodological considerations, in which no “time x maturity groups” interactions were found, acknowledging that both maturity groups benefited similarly from the training stimulus [51]. Conversely, these results conflict with LTAD theoretical position which advocate for an existing optimal window of opportunity for endurance development during or post-PHV [16]. Controversial results are shown on this topic: Baquet [53] observed similar peak VO_2_ improvements across maturational groups following intermittent endurance training. Our findings as well suggest that, within ecologically valid training environments such as SSGs, the physiological determinants of intermittent fitness remain highly trainable across a broader developmental range than traditionally proposed. Consequently, SSGs appear capable of eliciting meaningful aerobic adaptations regardless of players’ biological maturation.

An additional point worth reflecting on is whether the similar improvements in vIFT observed between maturity groups might be partly influenced by the unequal IL experienced during the intervention. Although EL was comparable, less mature players reported higher perceived exertion, suggesting differences in the relative physiological demand. This raises the question of whether more mature players might require a higher internal stimulus to fully exploit their adaptive potential, given their superior neuromuscular capacity and cardiovascular efficiency [54]. This interpretation is consistent with the principle that training adaptations are driven primarily by the magnitude of physiological stress [55]. Future research could examine whether equalizing IL through adjustments in task constraints would lead to different patterns of adaptation across maturity groups. Such inquiry may help clarify whether the absence of a maturity x time interaction reflects true similarity in trainability or simply the result of differing relative physiological stress.

For a broader understanding of these results, future research should investigate the underlying mechanisms that drive these adaptations, such as changes in muscle fiber type and neuromuscular efficiency, which lack an established consensus in the scientific literature. Larger sample sizes and longitudinal designs may help improve detection of maturational interactions, addressing the limitations of the present study. Although the intervention was integrated into the teams’ regular training schedules, the football-specific content of the weekly sessions was not strictly controlled, which may have introduced uncontrolled variability in the players’ overall training stimulus. Furthermore, including direct assessments of cardiorespiratory fitness (e.g., VO_2_max) together with the 30–15 IFT would help determine whether the improvements observed are primarily driven by changes in running speed or by more global metabolic adaptations. This approach would provide a more complete picture of how maturity status influences training responses in youth football players.

## 5. Conclusions

This study demonstrated that an 8-week training program based on SSG effectively improved vIFT in youth soccer players, regardless of their biological maturation status. Despite clear baseline differences in physical performance favoring players with advanced maturity, both maturity groups achieved similar relative improvements following the intervention. These findings suggest that the physiological mechanisms underpinning adaptations to intermittent, game-based training may be highly trainable across different maturational stages. Moreover, players with lower biological maturity perceived higher IL even when external demands were equivalent to their more mature peers. Overall, the findings emphasize that SSGs are an effective strategy to develop intermittent running fitness in youth soccer players, but their design and implementation should be carefully modulated based on player maturity, training objectives, and desired physical outcomes.

## Figures and Tables

**Table 1:** Descriptive data (M ± SD) and means comparison of physical performance variables according to the players’ maturity groups in the AS1.

Variable	MG1	MG2	t	ES (95%CI)
5 m sprint (s)	1.07 ± 0.09	1.01 ± 0.05	3.16 *	0.90 (0.31; 1.49)
30 m sprint (s)	4.67 ± 0.36	4.35 ± 0.25	3.70 *	1.06 (0.45; 1.65)
vIFT (km·h^−1^)	18.08 ± 1.47	19.37 ± 1.08	−3.63 *	−1.01 (−1.58; −0.42)

vIFT: final velocity in the 30–15 intermittent fitness test; MG1: maturity group 1; MG2: maturity group 2; * statistically different (*p* < 0.05); ES: effect size; t: t-value obtained from the independent samples *t*-test; CI: 95% confidence interval of the effect size.

**Table 2:** Descriptive data and comparison between EC and CG.

Variable	EG	CG	t	ES (95%CI)
Age (y)	14.25 ± 0.67	14.02 ± 1.21	0.88	0.25 (−0.31; 0.81)
PHV (y)	0.53 ± 0.86	0.19 ± 1.20	1.21	0.35 (−0.22; 0.91)
Body mass (kg)	58.80 ± 10.49	54.12 ± 10.99	1.54	0.44 (−0.13; 1.00)
Body height (cm)	168.44 ± 8.35	164.84 ± 7.34	1.58	0.45 (−0.12; 1.01)
5 m sprint (s)	1.04 ± 0.05	1.04 ± 0.10	0.12	0.03 (−0.54; 0.60)
30 m sprint (s)	4.48 ± 0.23	4.53 ± 0.46	−0.52	−0.15 (−0.72; 0.42)
vIFT (km·h^−1^)	18.92 ± 1.28	18.48 ± 1.63	1.10	0.31 (−0.25; 0.87)

vIFT: final velocity in the 30–15 intermittent fitness test; EG: experimental group; CG: control group; PHV: peak height velocity; ES: effect size; t: t-value obtained from the independent samples *t*-test; CI: 95% confidence interval of the effect size.

**Table 3:** Load differences between different SSG training tasks.

Measure	SSSG	LSSG	t	ES (95%CI)
Total distance (m)	1473.56 ± 128.05	1379.09 ± 176.64	4.63 *	0.74 (0.38; 1.09)
Walking (m)	532.80 ± 59.21	536.63 ± 72.08	−0.42	−0.07 (−0.38; 0.25)
LI running (m)	703.99 ± 119.29	612.88 ± 157.69	6.37 *	1.02 (0.63; 1.40)
MI runs (m)	175.92 ± 43.95	173.22 ± 49.91	0.40	0.06 (−0.25; 0.38)
HI runs (m)	3.47 ± 3.09	4.33 ± 3.82	−1.88	−0.30 (−0.62; 0.02)
Max. speed (km·h^−1^)	20.30 ± 1.39	20.36 ± 2.04	−0.19	−0.03 (−0.34; 0.28)
Total ball actions (n)	16.37 ± 13.20	8.31 ± 2.78	3.79 *	0.61 (0.26; 0.95)
LI ball actions (n)	6.85 ± 7.82	2.70 ± 1.16	3.26 *	0.52 (0.18; 0.85)
MI ball actions (n)	6.39 ± 3.34	3.43 ± 1.45	5.80 *	0.93 (0.55; 1.30)
HI ball actions (n)	3.15 ± 2.83	2.20 ± 1.29	2.22 *	0.36 (0.03; 0.68)
Shot speed (km·h^−1^)	39.23 ± 6.18	37.60 ± 6.82	1.22	0.20 (−0.12; 0.51)
Dribbling distance (m)	26.23 ± 23.51	19.60 ± 16.58	2.03 *	0.33 (0.00; 0.65)
HI accelerations (m)	44.07 ± 12.68	34.06 ± 10.57	6.05 *	0.97 (0.58; 1.35)
HI decelerations (m)	60.58 ± 11.70	48.44 ± 12.20	6.35 *	1.02 (0.62; 1.40)
MI accelerations (m)	99.97 ± 19.39	73.52 ± 20.21	7.89 *	1.26 (0.84; 1.68)
MI decelerations (m)	119.45 ± 21.54	93.48 ± 21.57	9.36 *	1.50 (1.03; 1.95)
RPE (0–10)	5.96 ± 0.95	5.51 ± 0.91	4.41 *	0.71 (0.35; 1.05)

SSSG: small small-sided games; LSSG: large small-sided games; LI: low-intensity; MI: medium-intensity; HI: high-intensity; RPE: rating of perceived exertion; * statistically different (*p* < 0.05); ES: effect size; t: t-value obtained from the paired-sample *t*-test; CI: 95% confidence interval of the effect size.

**Table 4:** Load differences between players with different maturity status.

Variable	MG1	MG2	t	ES (95% CI)
SSSG				
Total distance (m)	1475.18 ± 134.52	1408.63 ± 137.70	1.74	0.49 (−0.07; 1.04)
Walking (m)	526.54 ± 65.59	535.18 ± 55.63	−0.52	−0.15 (−0.70; 0.41)
LI running (m)	700.71 ± 149.37	639.70 ± 111.21	1.70	0.48 (−0.08; 1.03)
MI running (m)	186.60 ± 45.05	174.27 ± 39.29	1.05	0.30 (−0.26; 0.85)
HI running (m)	3.56 ± 2.10	4.65 ± 3.90	−1.17	−0.33 (−0.88; 0.23)
Max. speed (km·h^−1^)	20.30 ± 0.96	20.61 ± 1.69	−1.12	−0.32 (−0.87; 0.24)
Total ball actions (n)	12.83 ± 3.28	11.31 ± 7.44	0.88	0.25 (−0.31; 0.80)
LI ball actions (n)	4.85 ± 1.73	4.24 ± 4.30	0.61	0.17 (−0.38; 0.72)
MI ball actions (n)	5.40 ± 1.32	4.32 ± 2.11	2.09 *	0.59 (0.02; 1.15)
HI ball actions (n)	2.61 ± 1.42	2.77 ± 1.78	−0.35	−0.10 (−0.65; 0.46)
Shot speed (km·h^−1^)	38.50 ± 4.56	38.74 ± 4.54	−0.19	−0.05 (−0.60; 0.50)
Dribbling distance (m)	27.80 ± 18.88	18.85 ± 16.38	1.83	0.51 (−0.05; 1.07)
HI accelerations (m)	40.59 ± 8.53	38.25 ± 10.39	0.86	0.24 (−0.31; 0.79)
HI decelerations (m)	54.50 ± 10.00	55.97 ± 10.13	−0.52	−0.15 (−0.70; 0.41)
MI accelerations (m)	85.34 ± 13.36	91.07 ± 18.21	−1.24	−0.35 (−0.90; 0.21)
MI decelerations (m)	108.21 ± 19.91	108.82 ± 21.77	−0.10	−0.03 (−0.58; 0.52)
RPE (0–10)	6.07 ± 0.91	5.21 ± 0.62	4.11 *	1.16 (0.56; 1.74)
LSSG				
Total distance (m)	1418.91 ± 154.45	1366.50 ± 191.26	1.05	0.30 (−0.26; 0.85)
Walking (m)	530.59 ± 81.35	534.61 ± 67.85	−0.20	−0.06 (−0.61; 0.50)
LI running (m)	651.54 ± 178.00	592.71 ± 131.07	1.39	0.39 (−0.17; 0.94)
MI running (m)	179.47 ± 41.53	180.01 ± 57.38	−0.04	−0.01 (−0.56; 0.54)
HI running (m)	4.19 ± 2.62	5.04 ± 4.77	−0.75	−0.21 (−0.76; 0.34)
Max. speed (km·h^−1^)	20.36 ± 1.21	20.55 ± 2.45	−0.34	−0.10 (−0.65; 0.46)
Total ball actions (n)	9.32 ± 2.47	7.59 ± 3.06	2.17 *	0.61 (0.04; 1.17)
LI ball actions (n)	3.01 ± 1.25	2.38 ± 1.07	1.94	0.54 (−0.02; 1.10)
MI ball actions (n)	4.04 ± 1.24	2.99 ± 1.44	2.74 *	0.77 (0.20; 1.34)
HI ball actions (n)	2.31 ± 1.17	2.25 ± 1.28	0.19	0.05 (−0.50; 0.60)
Shot speed (km·h^−1^)	37.99 ± 4.77	37.93 ± 7.21	0.04	0.01 (−0.54; 0.56)
Dribbling distance (m)	26.55 ± 20.46	13.06 ± 10.16	3.19 *	0.90 (0.32; 1.47)
HI accelerations (m)	36.40 ± 9.37	32.33 ± 9.80	1.50	0.42 (−0.14; 0.98)
HI decelerations (m)	48.30 ± 11.23	49.31 ± 13.47	−0.29	−0.08 (−0.63; 0.47)
MI accelerations (m)	71.39 ± 11.48	77.21 ± 27.13	−0.93	−0.26 (−0.81; 0.29)
MI decelerations (m)	93.03 ± 16.99	95.71 ± 28.03	−0.39	−0.11 (−0.66; 0.44)
RPE (0–10)	5.88 ± 0.92	4.96 ± 0.68	4.18 *	1.18 (0.57; 1.77)

SSSG: small small-sided games; LSSG: large small-sided games; MG1: maturity group 1; MG2: maturity group 2; LI: low-intensity; MI: medium-intensity; HI: high-intensity; RPE: rating of perceived exertion; * statistically different (*p* < 0.05); ES: effect size; t: t-value obtained from the independent samples *t*-test; CI: 95% confidence interval of the effect size.

**Table 5:** Pairwise differences (pre–post) for each maturity group.

Variable		Pre	Post	t	ES (95% CI)
5 m sprint (s)	MG1	1.05 ± 0.07	1.05 ± 0.08	−0.09	−0.02 (−0.77; 0.72)
	MG2	1.03 ± 0.04	1.05 ± 0.06	−0.83	−0.21 (−0.91; 0.49)
30 m sprint (s)	MG1	4.53 ± 0.23	4.55 ± 0.26	−0.70	−0.08 (−0.39; 0.24)
	MG2	4.46 ± 0.23	4.46 ± 0.24	−0.19	−0.02 (−0.31; 0.27)
vIFT (km·h^−1^)	MG1	18.36 ± 1.22	19.43 ± 0.90	−3.14 *	−0.93 (−1.82; −0.05)
	MG2	19.32 ± 1.21	20.38 ± 1.21	−3.42 *	−0.92 (−1.74; −0.11)

vIFT: final velocity in the 30–15 intermittent fitness test; MG1: maturity group 1; MG2: maturity group 2; * statistically different (*p* < 0.05); ES: effect size; t: t-value obtained from the pairwise comparisons (post hoc) of RM ANOVA; CI: 95% confidence interval of the effect size.

## Data Availability

The dataset used in the present study is not available in a public repository but may be obtained from the corresponding author upon reasonable request. The data will be available for a period of three years following the publication of the article. Requests should be submitted via email to the corresponding author and must include a clear explanation of the purpose and intended use of the data.
